# Neuro-Ophthalmic Dengue Infection: A Case Report with a Multiple Body Site Sampling Strategy and Review of Laboratory Data

**DOI:** 10.3390/v16070998

**Published:** 2024-06-21

**Authors:** Grace I. Butel-Simoes, Nupur Bajaj, Sultan Asad, Jean Moselen, Nicole Orlando, Eike Steinig, Thomas Tran, Julian Druce, Leon Caly, Emma Bishop, Chanad Harangozo, Chuan Kok Lim

**Affiliations:** 1 Victorian Infectious Diseases Reference Laboratory, Royal Melbourne Hospital, Doherty Institute for Infection and Immunity, Melbourne, VIC 3000, Australia; 2 Department of Infectious Diseases, Peninsula Health, Melbourne, VIC 3199, Australia; 3 Department of Infectious Diseases, Doherty Institute of Infection and Immunity, The University of Melbourne, Melbourne, VIC 3000, Australia

**Keywords:** dengue virus, mosquito-borne encephalitis, uveitis, optic neuropathy, neuropathogenic virus

## Abstract

Dengue neurological disease is an uncommon yet severe complication of dengue infection. It can manifest as encephalitis, encephalopathy, neuro-ophthalmic complications, or neuromuscular disorders. Severe infection can result in viral shedding across multiple body sites. We describe a case of severe neuro-ophthalmic dengue infection in an otherwise healthy returned traveller, presenting with prolonged multiple-body-site viral detections by PCR. The dengue virus (DENV) dynamics and serological response support a direct DENV neuropathogenicity. A retrospective review of the laboratory data at the Victorian Infectious Diseases Reference Laboratory (VIDRL) suggests that blood is the most frequent sample type with DENV detection (92% of all DENV-positive samples). Genotype variation is seen across different sample types. The similarity of CSF and nasopharyngeal DENV subtypes (genotype 1 and 3) suggests a possible correlation between nasopharyngeal replication and neurological complications. The case presented highlights the direct neuropathogenicity of DENV early in the course of infection, and a potential correlation between nasopharyngeal replication and neurological disease.

## 1. Introduction

Dengue virus (DENV) is a single-stranded RNA *flavivirus* transmitted by mosquito vectors *Aedes aegypti* and *Aedes albopictus*. There are four genotypes of the virus, which circulate in tropical and subtropical endemic countries [1]. DENV causes a wide array of clinical manifestations, from asymptomatic to severe-end organ complications, such as severe neurological and ocular complications [2,3].

Prompt and accurate diagnosis is important for patient management, public health, and disease surveillance [1]. While clinical diagnosis of DENV may be possible, it can be difficult to distinguish from other infections that co-circulate in regions where DENV is found. Laboratory diagnosis commonly depends on the detection of the NS1 antigen, serology, and nucleic acid detection using molecular methods, such as a polymerase chain reaction (PCR) [1,4]. Virus culture and genome sequencing are infrequently performed for the purpose of primary diagnosis [4]. The timing of patient sample collection and the type of sample impact on the choice of test performed, as the yield of direct viral detection decreases as time from illness onset increases, and sample performance varies [1,4].

We report a case of severe neuro-ophthalmic dengue infection in a healthy person and the comprehensive laboratory testing performed. We describe the microbiology testing used to diagnose and study the viral dynamics in this patient with prolonged shedding. Additionally, reference laboratory data from the Victorian Infectious Diseases Reference Laboratory (VIDRL) on dengue virus molecular testing are also presented to assess the yield of multi-body-site sampling.

## 2. Case Report

We report a case of severe neurological dengue infection from an immunocompetent 65-year-old female. Upon returning to Australia from a trip to Bali four days prior, she presented to an outer metropolitan hospital in Victoria, Australia, with a ten-day history of febrile illness. Initially, she experienced diarrhoea and nausea whilst overseas. On day six of being unwell, the same day she returned to Australia, she developed fatigue, headaches, myalgias, and mild confusion.

On day three of her admission, the patient developed worsening headaches and confusion, now associated with photophobia, periorbital pain, and neck stiffness. A cranial nerve examination revealed binocular diplopia and a visual acuity of 6/15. Fundoscopic ophthalmic examination and retinal fibre thickness assessment by optical coherence tomography revealed features of bilateral non-arteritic ischaemic optic neuropathy and bilateral acute anterior uveitis. She was commenced on topical predneferin forte eye drops and completed a two-week course of treatment.

A peripheral blood examination showed thrombocytopenia (108 × 10^9^/L) with reactive erythrocytes on blood film, a normal white cell count (3.5 × 10^9^/L), and CRP (5 mg/L). Renal and liver functions were slightly deranged. (Creatinine 89 μmol/L, ALT 65 units/L, AST 72 units/L, GGT 171 units/L, ALP 190 units/L, INR 0.9.) She tested positive for norovirus via faecal PCR testing. Neuroimaging, including brain computed tomography (CT) scanning and brain magnetic resonance imaging (MRI), were unremarkable. Lumbar puncture examination demonstrated normal cerebrospinal fluid (CSF) parameters: glucose 4.0 mmol/L (range: 2.0–3.9 mmol/L), protein 0.42 g/L (range: 0.15–0.45), white blood cells 3 × 10^6^/L, and erythrocytes 0 × 10^6^/L. No organisms were seen on gram-stain and routine bacterial and fungal cultures were negative. PCR for herpes viruses (herpes simplex virus 1 and 2, varicella zoster virus), enterovirus, and endemic flaviviruses (Japanese encephalitis virus, Murray valley encephalitis virus, Kunjin virus, and Zika virus), as well as cryptococcal antigen testing on the CSF, were all negative.

### 2.1. Multisite Sampling and Laboratory Finding

**Figure 1:**
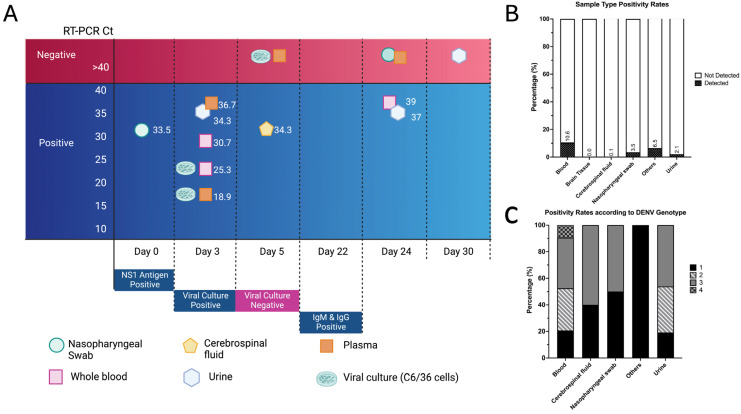
Temporal viral dynamics of the DENV encephalitis case and retrospective laboratory data review. (**A**) Dengue virus (DENV) detection (RT-PCR) and isolation from case diagnosis. The *Y*-axis indicates the DENV RT-PCR cycle threshold (Ct), and the numbers associated with each symbol indicate the Ct of the clinical samples. The *X*-axis indicates the days from admission. Viral culture was performed on three samples with a successful isolation of DENV on day 3 and a negative culture on day 5. This figure was created using Biorender. (**B**) A retrospective review of DENV RT-PCR results (years 2010–2024), demonstrating percentage positivity by sample types. Annotations within the graph indicate detection percentage. (**C**) Percentage distribution of DENV genotype, according to sample types. Labels on the right (1–4) indicate the DENV subtypes.

Despite a negative MRI and normal CSF, neurological dengue infection was suspected due to recent travel history. Whole blood and plasma were sent to the Victorian Infectious Diseases Reference Laboratory, and a diagnosis of dengue virus (DENV) infection was made by the detection of dengue virus Type 1 (DENV-1) with pan-flavivirus and dengue virus real-time PCR (RT-PCR) [5,6]. Additional clinical samples collected around the time of presentation, including cerebrospinal fluid, urine, and a nasopharyngeal swab, were all positive by DENV RT-PCR. (Figure 1A) A primary DENV infection was confirmed, with the presence of NS1 Ag detection in combination with negative IgM and IgG antibodies in early acute serology (PlateliaTM Dengue NS1Ag, Biorad, France and Panbio Dengue IgM capture and IgG Indirect ELISA, Alere) [7]. The virus was successfully isolated from the patient’s blood (whole blood and plasma) using C6/36 and Vero cells as previously described [6]. Virus isolations from CSF and urine were not successful.

Due to ongoing symptoms, serial testing of blood, urine, and nasopharyngeal swabs for DENV-1 RNA were performed over the month, with DENV-1 detected by PCR for up to 24 days in urine and whole blood since the initial diagnosis. (Figure 1A) Convalescent sera collected 22 days later showed IgG seroconversion and the persistence of IgM in keeping with a recent infection.

### 2.2. Serological and Genotyping Confirmation

A viral neutralisation test (VNT) was performed for the purpose of (1) as an adjunct to molecular subtyping and (2) for determining the capability of the patient to mount an effective humoral response. As previously described, following the inoculation of serum into Vero cells, the culture supernatant was harvested at 48 h post-infection and assayed by RT-PCR to provide a more objective read out [8]. The patient serum collected at day 22 demonstrated a presence of antibodies consistent with a neutralising titre of 160 to DENV-1. Neutralising antibodies were not detected in the CSF at day 5.

Whole-genome sequencing was performed directly from the whole-blood sample, using both tiled amplicon [9] (pan-dengue and dengue virus serotype 1) and hybrid-capture NGS (TWIST Bioscience, as previously described) [10], achieving close to whole-genome recovery (97.6% DENV-1 genome coverage).

## 3. Retrospective Review of Laboratory Data

The finding of multiple DENV-positive samples in this case, including uncommon sample types such as a nasophayngeal swab and CSF, prompted a laboratory look-back to assess the frequency of such results and to interrogate the yield of different sample types. A review of dengue PCR testing performed at VIDRL between 2010 and March 2024 was conducted to assess the yield of multi-body-site sampling strategies for acute dengue infection. In that period, a total of 8,042 samples were tested, and, of these, 476 (5.91%) had DENV detected. The majority of DENV detection derives from blood samples (92%, n = 439/476), followed by urine (6%, n = 27/476), CSF (1%, n = 3/476), and nasopharyngeal swabs (0.8%, n = 4/476). The respective frequencies of DENV detection are 10.5% for blood (n = 439/4161), 3.5% for nasopharyngeal swabs (n = 4/115), 2.1% for urine (n = 27/1289), and 0.1% for CSF (n = 3/2178). No DENV was detected from brain tissue (n = 253), and a 6.5% detection rate was observed among miscellaneous samples (n = 3/46) (Figure 1B). For the selection of samples where the Ct value was available, the mean Ct for blood is 30.86 (95% CI 30.34–31.38, n = 146) and urine 36.4 (95%CI 36.12–36.72, n = 12). There were insufficient CSF samples to determine the mean Ct.

Dengue virus subtype 3 (DENV-3) was the most commonly detected genotype (38.65%), followed by DENV-2 (31.44%), DENV-1 (21.18%), and DENV-4 (8.73%) (Figure 1C). Multiple DENV subtypes were seen in both blood and urine (blood DENV-1–4; urine DENV-1–3), whereas DENV-1 and DENV-3 account for all almost equal proportions in CSF (40% vs. 60%, respectively) and NP swab (50% vs. 50%, respectively) detections.

## 4. Discussion

Dengue neurological disease can manifest as encephalitis, encephalopathy, neuro-ophthalmic involvement, or neuromuscular disorders, reported at a frequency of around 0.5% [1,11,12,13,14]. Treatment is largely supportive, as the development of effective antivirals has been hindered by a lack of in vivo models that replicate severe dengue infection [15]. The prognosis for neurological disease is variable [9], and predictor for disease development remains elusive. Our case highlights that dengue neurological disease can occur in a healthy individual capable of mounting an effective immunological response, and the early neurological manifestation is probably a result of direct viral neurological pathogenicity. This is supported by the in vivo modelling of neuroinvasiveness, neurotropism, and neurovirulence in DENV [10].

Previous reports suggest that dengue neurological disease is most commonly attributed to DENV-2 and DENV-3 [16,17], although in vitro or in vivo study to support enhanced neurotropism in these DENV subtypes is lacking. One study examining DENV whole-genome sequences derived from corresponding blood and CSF showed high similarities between the sequences, suggesting that the pathogenesis of neurological dengue is a direct breach of the blood–brain barrier in the event of DENV viraemia [18]. In our retrospective review, DENV-1 and DENV-3 are the most common subtypes detected in CSF. Similar subtypes are also seen in NP swabs, raising the possible correlation between nasopharyngeal viral replication and neurological dengue infection. In contrast, multiple DENV subtypes can be detected in blood and urine, suggesting that urine DENV excretion is a direct reflection of viraemia.

Compared with previous studies, prolonged viremia (median of 30 days) is not uncommon in immunocompromised patients [19]. However, such prolonged viraemia (24 days post admission) in an immunocompetent individual is very unusual and is significantly out of proportion to the expected duration, even for a primary infection (ranging from 4 to 14 days) [15]. The exact duration of DENV symptoms in this case was difficult to delineate, due to the concurrent norovirus infection. We suspect that on the day of hospital admission, the patient was on day four of dengue illness. The duration of PCR positivity presented is calculated from the day of admission and is likely an underestimate of the exact duration. It is worth noting that the detection of DENV RNA through nucleic acid testing was positive for a prolonged period; however, viral culture was only successful on the early sample. This casts doubt on whether there was ongoing viral replication of competent virus or just persistence of RNA.

Our retrospective review suggests that blood samples have the highest yield compared to other sample types, consistent with previous reports [19]. However, the disease prognostic value for the duration of viraemia and viruria is currently unknown. DENV detection in urine can be prolonged and may exceed the viraemia period, and hence could be a useful supplementary sample for DENV genotyping in late presenters [10,20,21].

## 5. Conclusions

We present a case of severe neuro-ophthalmic dengue infection in an immunocompetent patient with prolonged virus shedding across multiple body sites. The virus dynamics and serological response support a direct DENV neuropathogenicity. This retrospective review of laboratory data suggests blood is the most frequent sample type with DENV detection. The similarity of CSF and nasopharyngeal DENV subtypes suggests a possible correlation between nasopharyngeal replication and neurological complications. This case highlights (1) the direct neuropathogenicity of DENV early in the course of infection, and (2) the potential correlation between nasopharyngeal replication and neurological disease. Finally, this case underlines the need for DENV direct antiviral development for severe neurological disease.

## Data Availability

Laboratory audit data has been provided in the article, further inquiries can be directed to the corresponding author.
